# Supplementary material for: DMRT1-mediated reprogramming drives development of cancer resembling human germ cell tumors with features of totipotency
Source: Nat Commun. 2021 Aug 19;12:5041. doi: 10.1038/s41467-021-25249-4 (PMC8377058; doi:10.1038/s41467-021-25249-4)
Supplement: Supplementary file 1 — Supplementary Information [file 41467_2021_25249_MOESM1_ESM.pdf]

Supplementary Information

DMRT1-mediated reprogramming drives development of cancer resembling human germ cell tumors with features of totipotency

Taguchi et al.

Inventory of Supplementary Information

Supplementary Figures (Supplementary Figures 1-10)
Legends to Supplementary Figures
Reference
Supplementary Table S1

Supplementary Figure 1

a

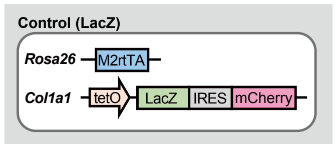

b

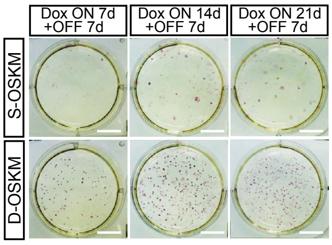

c

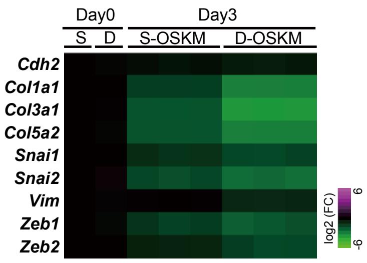

d

Day 14 D-OSKM MEFs upregulated genes (n=778)

GO ACCESSION	GO Term (BP)	p-value
GO:0030216	keratinocyte differentiation	1.68E-11
GO:0008544	epidermis development	6.60E-11
GO:0031424	keratinization	1.18E-10
GO:0018149	peptide cross-linking	1.67E-08
GO:0070830	bicellular tight junction assembly	1.33E-06
GO:0016338	calcium-independent cell-cell adhesion	7.01E-06
GO:0043588	skin development	1.33E-05
GO:0061436	establishment of skin barrier	1.96E-05
GO:0045071	negative regulation of viral genome replication	4.59E-04
GO:0003334	keratinocyte development	5.83E-04

Day 14 D-OSKM MEFs downregulated genes (n=289)

GO ACCESSION	GO Term (BP)	p-value
GO:0006935	chemotaxis	2.11E-10
GO:0006954	inflammatory response	5.67E-08
GO:0002376	immune system process	6.29E-08
GO:0030593	neutrophil chemotaxis	2.07E-06
GO:0009952	anterior/posterior pattern specification	1.03E-05
GO:0006955	immune response	3.47E-05
GO:0050729	positive regulation of inflammatory response	1.24E-04
GO:0045087	innate immune response	1.38E-04
GO:0008284	positive regulation of cell proliferation	1.42E-04
GO:0009954	proximal/distal pattern formation	2.72E-04

e

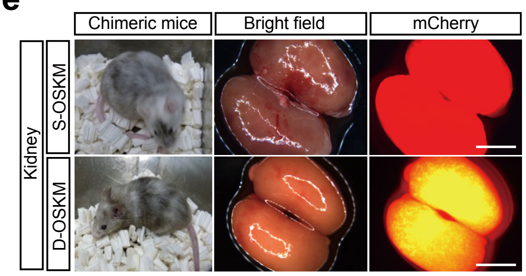

f

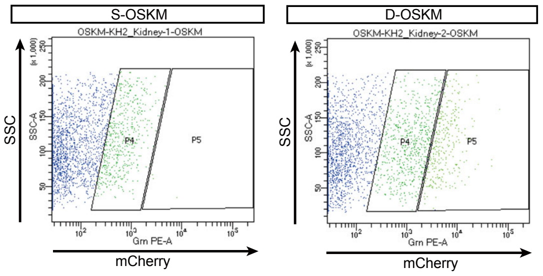

g

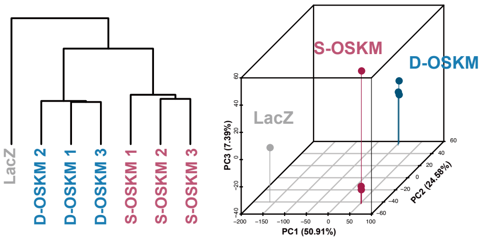

Supplementary Fig. 1: D-OSKM reprogramming *in vitro* and *in vivo*.

a, A schematic illustration of the genomic construct for Dox-inducible LacZ ESCs.
b, Alkaline phosphatase staining for S-/D-OSKM MEFs after Dox treatment for 7, 14 and 21 days, followed by the withdrawal for 7 days. Scale bars: 10 mm.
c, Heatmap showing relative expression of MEF-related genes in S-/D-OSKM MEFs at Day 3. Repression of MEF-related genes were prominent in D-OSKM MEFs when compared with S-OSKM MEFs, indicating the rapid repression in D-OSKM MEFs. Color range is shown using a $\log_2$ scale.
d, Gene ontology (GO) term enrichments of upregulated (left) and downregulated (right) genes in D-OSKM MEFs (Day 14) when compared with S-OSKM MEFs (Day 14) (Fold change>2, P<0.05). GO terms and the p-value are shown (Fisher's exact test, one-sided.). DAVID Bioinformatics Resources (v6.8) was used for the analysis.
e, Representative images showing mCherry signals in the kidney of S-/D-OSKM chimeric mice after Dox treatment for 10 days. Scale bars: 5 mm.
f, Flow cytometry analysis of kidney cells of S-/D-OSKM chimeric mice after Dox treatment for 7 days.
g, Un-supervised hierarchical clustering and PCA of transcriptomes of mCherry-positive *OSKM*-expressing kidney cells (Day 14).

Supplementary Figure 2

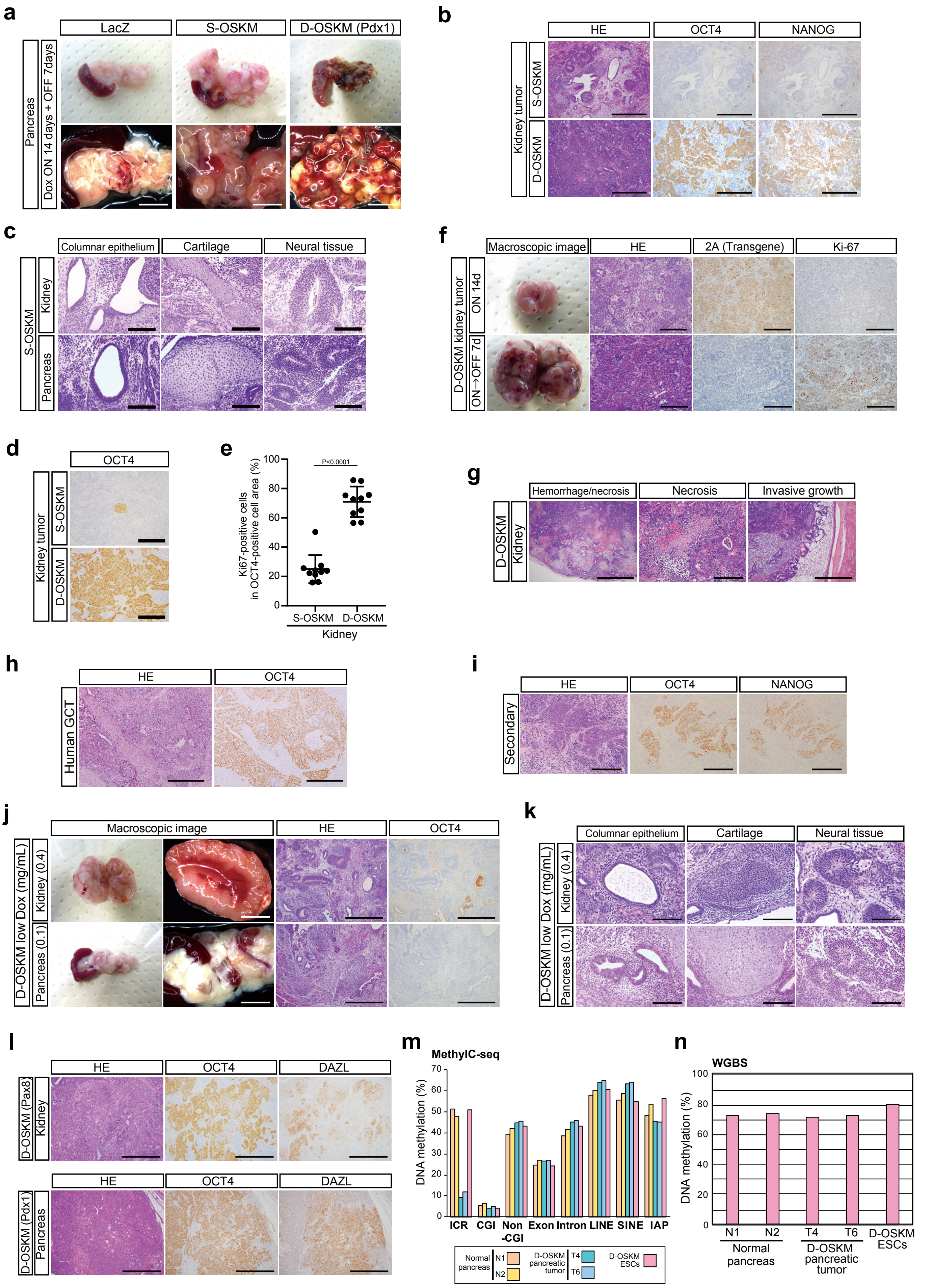

Supplementary Fig. 2: Development of D-OSKM tumors that resemble human germ cell tumors.

a, Representative macroscopic images of pancreatic tumors in S-/D-OSKM chimeric mice. Scale bars: 5 mm.

b, Representative histological images and immunostaining for OCT4 and NANOG of S-/D-OSKM kidney tumors. Scale bars: 500 μm.

c, Representative histological images of S-OSKM kidney and pancreatic tumors. Scale bars: 200 μm.

d, Immunostaining for OCT4 of S-/D-OSKM kidney tumors. Scale bars: 200 μm.

e, Quantification of the Ki67-positive cell ratio in the OCT4-positive cell area of S-/D-OSKM kidney tumors (n=10, respectively). The vast majority of OCT4-expressing D-OSKM tumor cells are positive for the cell proliferation marker Ki67, suggesting that the propagation of OCT4-expressing cells is responsible for tumor formation in the D-OSKM model. Data are presented as means ± SD of biologically independent samples. (Mann–Whitney test, two-sided.)

f, Representative macroscopic and histological images of the D-OSKM kidney. Active cell proliferation in D-OSKM tumors was initiated after withdrawal of Dox, indicating that exogenous OSKM expression was dispensable for active proliferation of tumor cells. Immunostaining for 2A peptide and Ki-67 is shown in the right panels. Expression of 2A peptide represents exogenous reprogramming factor expression. Scale bars: 200 μm.

g, Representative histological images of D-OSKM kidney tumors showing malignant features. Scale bars: 500 μm (left and right), 200 μm (middle).

h, Representative histological images and immunostaining for OCT4 of human embryonal carcinomas in the testis. Scale bars: 200 μm.

i, Representative histological images and immunostaining for OCT4 and NANOG of secondary D-OSKM tumors in the subcutaneous tissue of immunocompromised mice. Scale bars: 200 μm.

j, Representative macroscopic and histological images of D-OSKM tumors induced by low-dose Dox (kidney, 0.4 mg/mL; pancreas, 0.1 mg/mL). Scale bars: 5 mm (left), 500 μm (middle and right).

k, Representative histological images of D-OSKM tumors (teratomas) induced by low-dose Dox. Scale bars: 200 μm.

l, Representative histological images and immunostaining for OCT4 and DAZL of D-OSKM tumors. Scale bars: 500 μm.

m, DNA methylation levels at genomic elements in D-OSKM pancreatic tumors measured by MethylC-seq analysis. CGI: CpG island; Non-CGI: Non-CpG island.

n, Total DNA methylation percentage in D-OSKM pancreatic tumors measured by WGBS.

Supplementary Figure 3

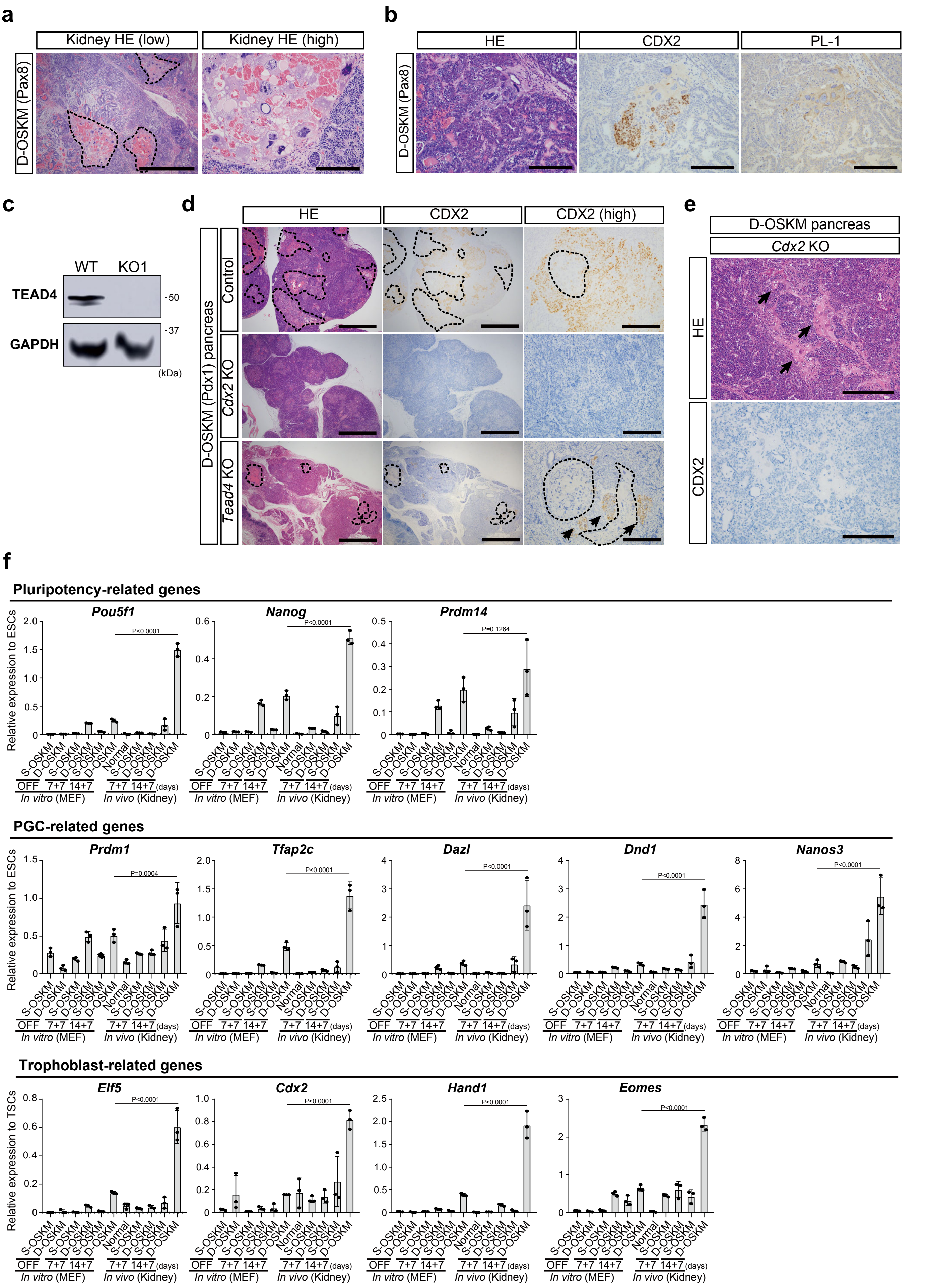

Supplementary Fig. 3: Trophoblastic differentiation of D-OSKM tumor cells.

a, Representative histological images of D-OSKM kidney tumors with TGC clusters. Dotted lines indicate the TGC clusters. Scale bars: 500 µm (left), 200 µm (right).

b, Representative histological images and immunostaining for CDX2 and PL-1 of D-OSKM kidney tumors. Scale bars: 200 µm.

c, Western blotting for TEAD4 in *Tead4* KO D-OSKM ESCs. Lack of CDX2 protein expression was confirmed by immunostaining of D-OSKM tumors (see Extended Data Fig. 3d).

d, Representative histological images and immunostaining for CDX2 of D-OSKM pancreatic tumors. The number of trophoblast giant cells was significantly decreased in both *Tead4* KO and *Cdx2* KO D-OSKM tumors. A small population of trophoblast giant cells was still detectable in *Tead4* KO D-OSKM tumors, indicating the presence of an alternative trophoblastic differentiation pathway in D-OSKM tumors. Dotted lines indicate the TGC clusters, and arrows indicate CDX2-positive cells. Scale bars: 1000 µm (left and middle), 200 µm (left panels: right).

e, A small population of trophoblast giant cells was still detectable in *Cdx2* KO D-OSKM tumors, indicative of the presence of an alternative trophoblastic differentiation pathway. Arrows indicate TGCs. Scale bars: 200 µm.

f, qRT-PCR analyses for expression of pluripotency-, PGC- and trophoblast-related genes during reprogramming *in vitro* (MEFs) and *in vivo* (kidney). Data are presented as the means ± SD of biological triplicates. Relative expression levels to ESCs are shown for pluripotency- and PGC-related genes. Relative expression levels to TSCs are shown for trophoblast-related genes. (one-way ANOVA and Dunnett's multiple comparisons test, two-sided.)

Supplementary Figure 4

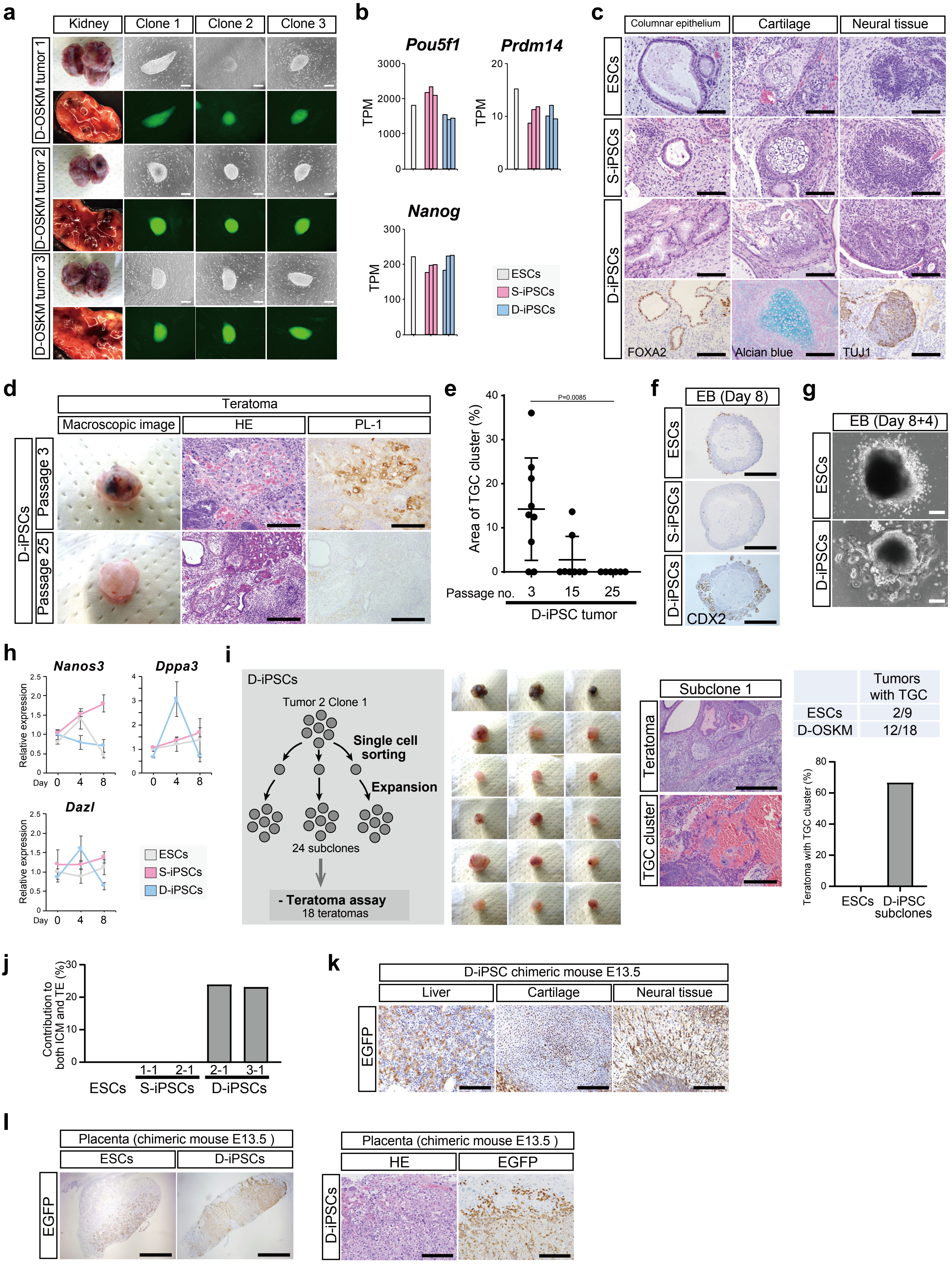

Supplementary Fig. 4: Derivation of D-OSKM tumor-derived PSCs with differentiation potential toward extra-embryonic lineage.

a, Representative images of D-OSKM kidney tumors and iPSC-like cell colonies derived from D-OSKM kidney tumor cells. A total of nine D-iPSC clones were established from three independent kidney tumors. Scale bars: 200 μm.

b, Expression levels of pluripotency-related genes in S-/D-iPSCs.

c, Representative histological images of subcutaneous tumors in immunocompromised mice. Scale bars: 200 μm.

d, Representative macroscopic and histological images of subcutaneous tumors developed from D-iPSCs with different passage numbers (passage number 3 and 25). Immunostaining for PL-1 is shown in the right panels. Scale bars: 200 μm.

e, Quantification of the TGC cluster area in D-iPSC tumors with different passage numbers (p3, n=9; p15, n=8; p25, n=6). Data are presented as means ± SD of biologically independent samples. (Kruskal–Wallis test and Dunn's multiple comparisons test, two-sided.)

f, CDX2 immunostaining of EBs at differentiation Day 8. Scale bars: 500 μm.

g, Representative images of EBs after attachment on the gelatin-coated dish. Scale bars: 200 μm.

h, qRT-PCR analyses for expression of PGC-related genes during EB formation. Data are presented as the means ± SD of biological triplicates. The mean expression level of differentiation Day 0 ESCs was set to 1.

i, A schematic illustration of an experimental protocol for D-iPSC subclones. The majority of D-iPSC subclone-derived tumors contain clusters of TGCs. Scale bars: 500 μm (upper), 200 μm (lower).

j, The frequency of blastocysts exhibiting GFP-labeled cells at both ICM and TE regions (ESCs, n=122; S-iPSCs 1-1, n=66; S-iPSCs 2-1, n=64; D-iPSCs 2-1, n=67; D-iPSCs 3-1, n=39).

k, GFP immunostaining of tissues in the E13.5 D-iPSC chimeric embryo. Scale bars: 200 μm.

l, GFP immunostaining of the E13.5 chimeric placenta. Scale bars: 1000 μm (left), 200 μm (right).

Supplementary Figure 5

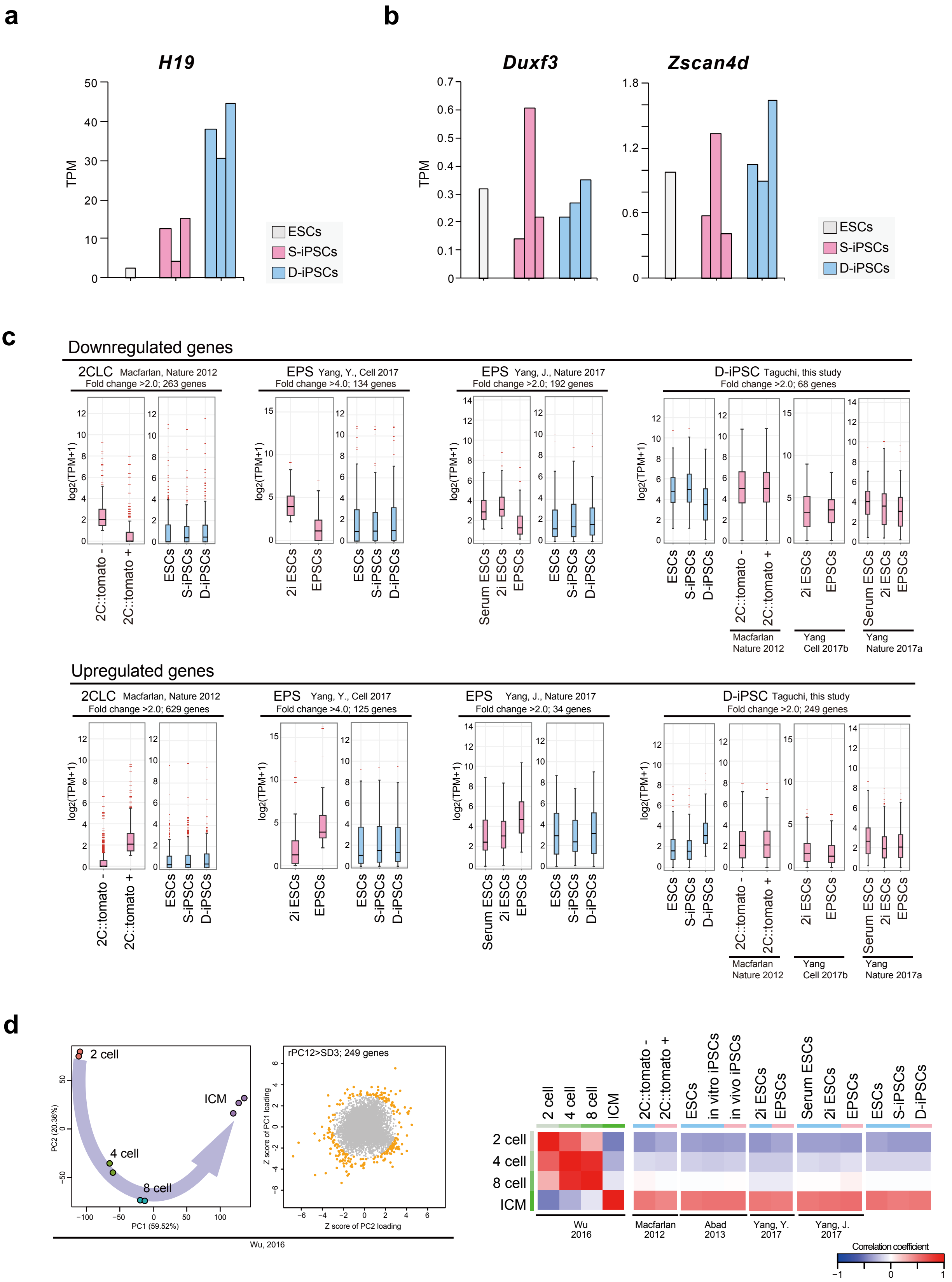

Supplementary Fig. 5: Gene expression profiles of D-iPSCs.

a, Expression levels of *H19* in ESCs, S-iPSCs, and D-iPSCs.
b, Expression levels of 2CLC-related genes in ESCs, S-iPSCs, and D-iPSCs.
c, Box plots for expression levels of upregulated/downregulated genes in each PSC type. Gene expression patterns of D-iPSCs were compared with previously reported mouse PSCs with expanded differentiation potential. Solid lines in each box indicate the median. The bottom and top of the boxes are lower and upper quartiles, respectively. Whiskers extend to ±1.5 interquartile range (IQR). RNA-seq data of PSCs were obtained from GSE33923, GSE89303, and ERP005641.
d, PCA of the preimplantation embryos by all expressed genes (upper, left) from GSE66390 [1]. Scatter plot of the normalized loading scores of PCA (upper, right). Orange dots (249 genes) indicate genes that contributed highly to the PC1 and PC2 axes: >3 s.d. radius of PC1 and PC2 (rPC12>SD3) [2]. Heatmap shows transcriptional correlation between preimplantation embryos and PSCs with expanded differentiation potential using the 249 genes from the upper panel [3-6]. RNA-seq data of PSCs were obtained from GSE33923, GSE48364, GSE89303, and ERP005641.

Supplementary Figure 6

a

	Total read number	Mapping efficiency (%)
ESCs (LacZ)	125,787,010	96.7
S-iPSC (p3)	117,389,850	94.5
D-iPSC (p3)	116,012,340	96.4
D-iPSC (p25)	141,428,232	96.3

	Common peaks	Differential peaks	
		Enriched in D-iPSC (p3)	Depleted in D-iPSC (p3)
vs ESCs (LacZ)	55,911	2,109	6,608
vs S-iPSC (p3)	56,078	1,860	6,623
vs D-iPSC (p25)	55,458	1,787	3,773
		Overlapping peaks 270	Overlapping peaks 992

b

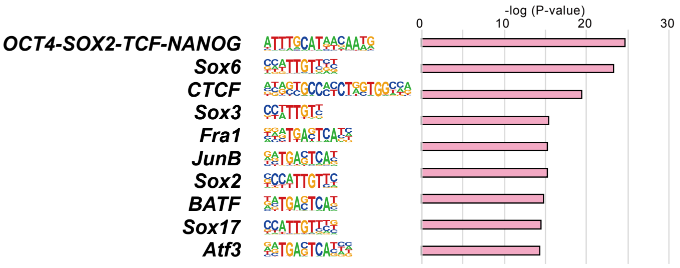

c

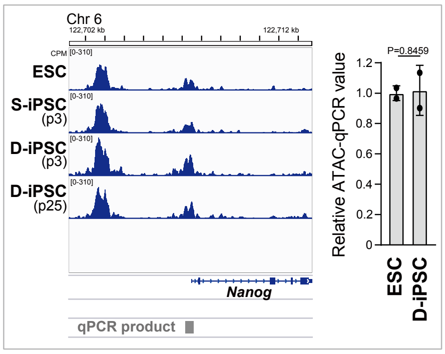

d

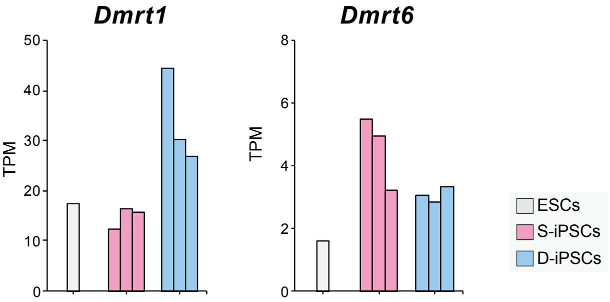

Supplementary Fig. 6: Elevated DMRT1 chromatin accessibility in D-PSCs.

a, (Upper) Total numbers of ATAC-seq sequenced reads and the mapping efficiencies (ESCs, S-iPSCs (p3), D-iPSCs (p3) and D-iPSCs (p25), n=1, respectively). (Lower) Common and differential peaks in D-iPSCs (p3) that have bidirectional differentiation potential in comparison with other PSCs.

b, Motif enrichment analysis of D-iPSC–specific depleted peaks in ATAC-seq (n=992), performed using HOMER (Fisher's exact test, two-sided).

c, The *Nanog* locus was used for control peaks in ATAC-qPCR analysis. A genomic region of qPCR products is shown below. Data are presented as means ± SD of biological duplicates. The mean ATAC-qPCR value of ESCs was defined as 1. (t-test, two-sided)

d, Expression levels of *Dmrt1* and *Dmrt6* in ESCs, S-iPSCs, and D-iPSCs.

Supplementary Figure 7

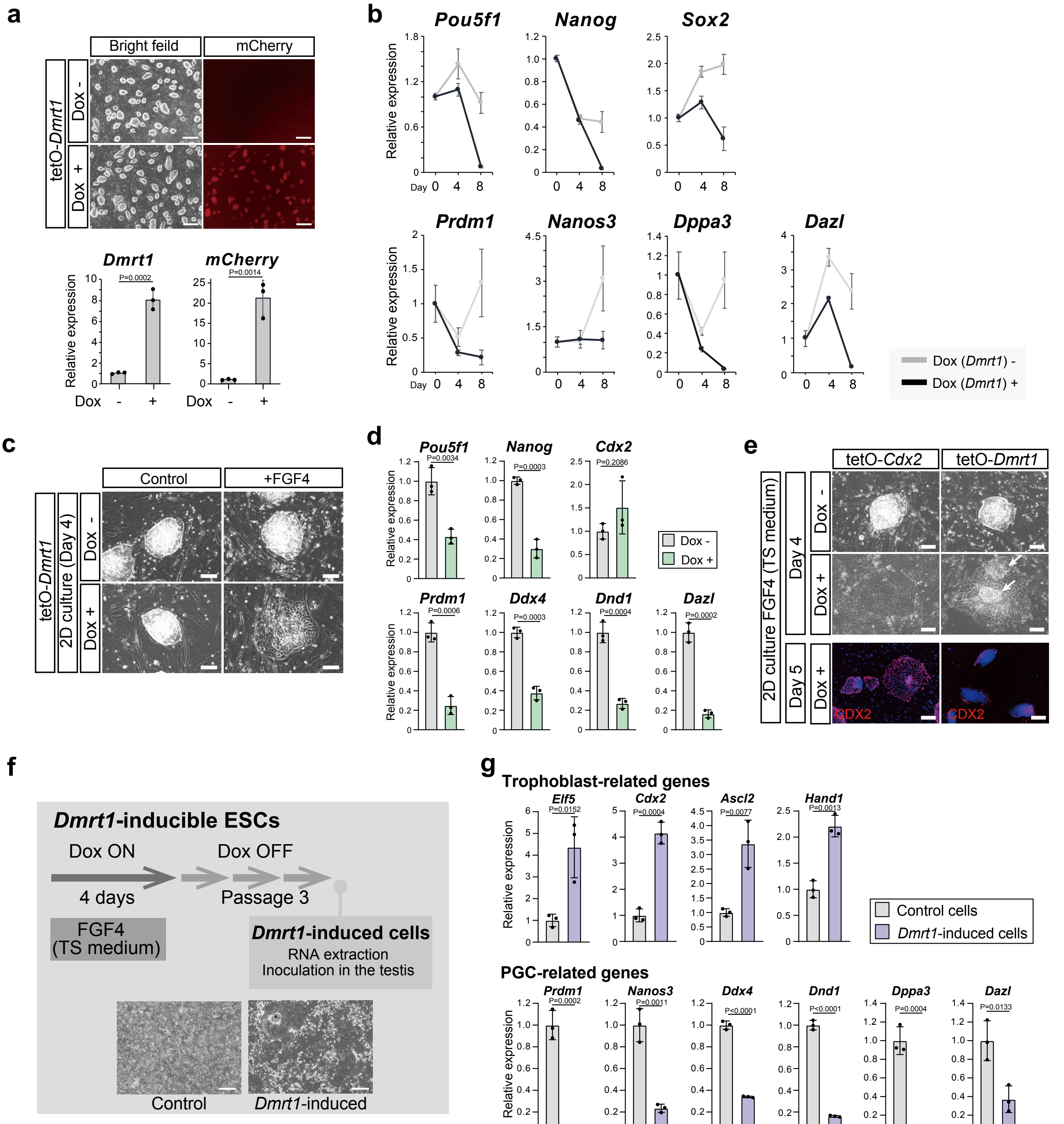

Supplementary Fig. 7: Elevated DMRT1 chromatin accessibility is associated with the expanded differentiation potential in PSCs.

a, Representative images and qRT-PCR analyses of Dox-inducible *Dmrt1* ESCs on Day 1 after Dox treatment. Scale bars: 500 μm. Data are presented as means ± SD of biological triplicates. Mean expression level of Dox (-) ESCs was defined as 1. (t-test, two-sided.)
b, qRT-PCR analyses of pluripotency- and PGC-related gene expression during EB differentiation of *Dmrt1*-inducible ESCs. Data are presented as means ± SD of biological triplicates. The mean expression level of Dox (-) ESCs was defined as 1.
c, Representative images of *Dmrt1*-inducible ESCs treated in FGF4-containing culture medium. Scale bars: 100 μm.
d, qRT-PCR analyses of pluripotency- and PGC-related gene expression in *Dmrt1*-inducible ESCs in FGF4-containing medium. Data are presented as means ± SD of biological triplicates. The mean expression level of ESCs at Day 0 was defined as 1. (t-test, two-sided.)
e, Representative images and CDX2 immunostaining of *Cdx2*- and *Dmrt1*-inducible ESCs in FGF4-containing medium (TS medium). Although *Cdx2* induction for 4 days caused rapid morphological changes in most ESCs, dome-shape colonies (arrows) were still present after *Dmrt1* induction for 4 days. Scale bars: 100 μm (bright field), 200 μm (CDX2).
f, Schematic illustration of the experimental protocol for transient induction of *Dmrt1*. Scale bars: 500 μm.
g, qRT-PCR analyses of trophoblast- and PGC-related gene expression in *Dmrt1*-induced cells. Data are presented as means ± SD of biological triplicates. The mean expression level of control cells was defined as 1. (t-test, two-sided.)

Supplementary Figure 8

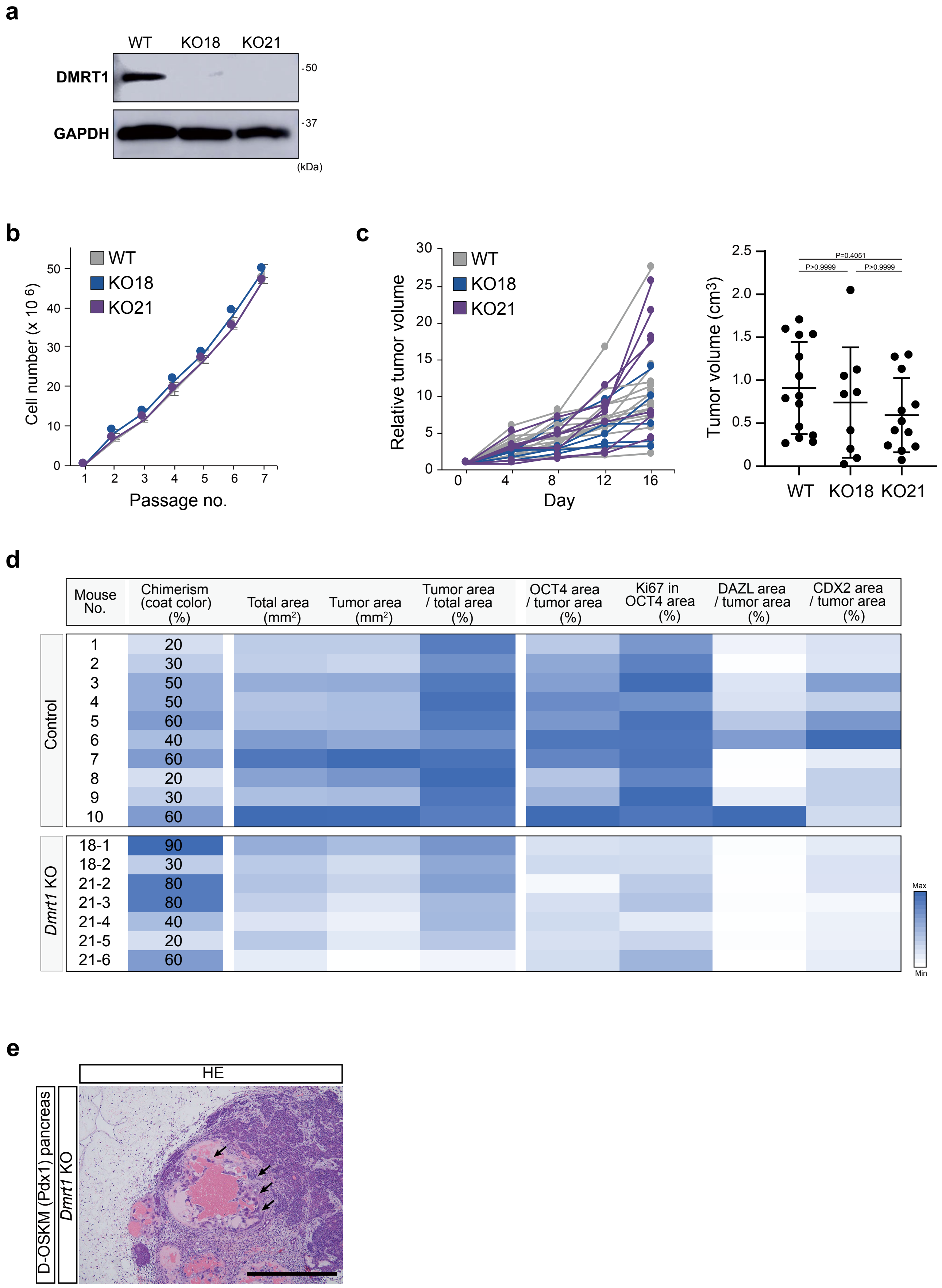

Supplementary Fig. 8: DMRT1 drives *in vivo* reprogramming and propagation of GCT-like tumor cells.

a, Western blotting for DMRT1 in *Dmrt1* KO D-OSKM ESCs.
b, Cell proliferation assay in *Dmrt1* KO D-OSKM ESCs. Data are presented as means ± SD of three independent experiments.
c, Tumor growth of *Dmrt1* KO D-OSKM ESCs in teratoma assay. Right panel represents tumor volume at Day 16 (control, n=14; *Dmrt1* KO ESCs [KO18, n=8; KO21, n=12]). Data are presented as means ± SD of biologically independent samples. (t-test with Welch's correction, two-sided.)
d, Coat color chimerism in each *Dmrt1* KO chimeric mouse used in this study. Quantification of histological analysis is summarized for each mouse that developed pancreatic tumors.
e, Histological image of *Dmrt1* KO D-OSKM pancreatic tumor. Although *Dmrt1* KO D-OSKM tumors exhibited a significant reduction in CDX2-positive cells, D-OSKM tumors still contained clusters of trophoblast giant cells (arrows), suggesting the presence of an alternative pathway for differentiation into trophoblast giant cells. Scale bars: 500 μm.

Supplementary Figure 9

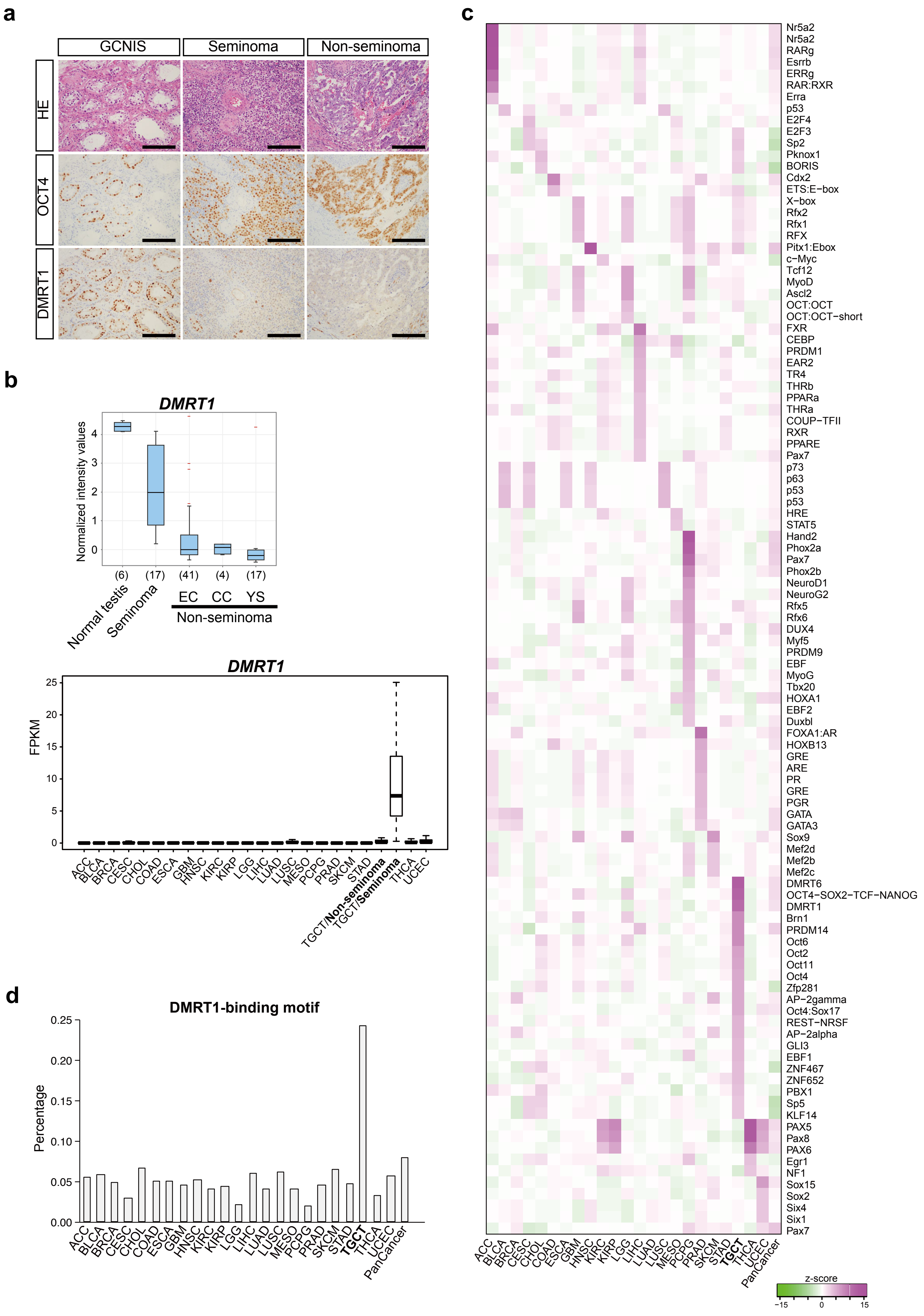

Supplementary Fig. 9: DMRT1-mediated reprogramming in human germ cell tumors.

a, Representative histological images and immunostaining for OCT4 and DMRT1 in human testicular neoplasms (GCNIS: Germ cell neoplasia in situ). Note that GCNIS exhibits the highest expression of DMRT1. Scale bars: 200 μm.

b, (Upper) Box plot showing expression of *DMRT1* in human testicular GCTs (normal testis, n=6; seminoma, n=17; non-seminoma [EC: embryonal carcinoma, n=41; CC: choriocarcinoma, n=4; YS: yolk sac tumor, n=17]). Microarray data were obtained from GSE3218 [7]. Solid lines in each box indicate the median. The bottom and top of the boxes are lower and upper quartiles, respectively. Whiskers extend to ±1.5 interquartile range (IQR).

(Lower) Box plot showing FPKM (expression levels) of DMRT1 in 23 types of human cancer. Solid lines in each box indicate medians. The bottom and top of the boxes indicate the lower and upper quartiles, respectively. Whiskers extend to ±1.5 interquartile range (IQR). Each cancer type expression dataset, obtained by RNA-seq, was obtained from the NIH GDC Data Portal (https://portal.gdc.cancer.gov/).

c, Heatmaps are shown as in Fig. 7b. Transcription factors are ordered based on the proportions of cancer type-specific open chromatin regions with their consensus motifs. The dataset from Fig. 7b was used.

d, Proportion of each cancer type-specific open chromatin region that contains DMRT1 consensus motifs. The dataset from Fig. 7b was used.

Supplementary Figure 10

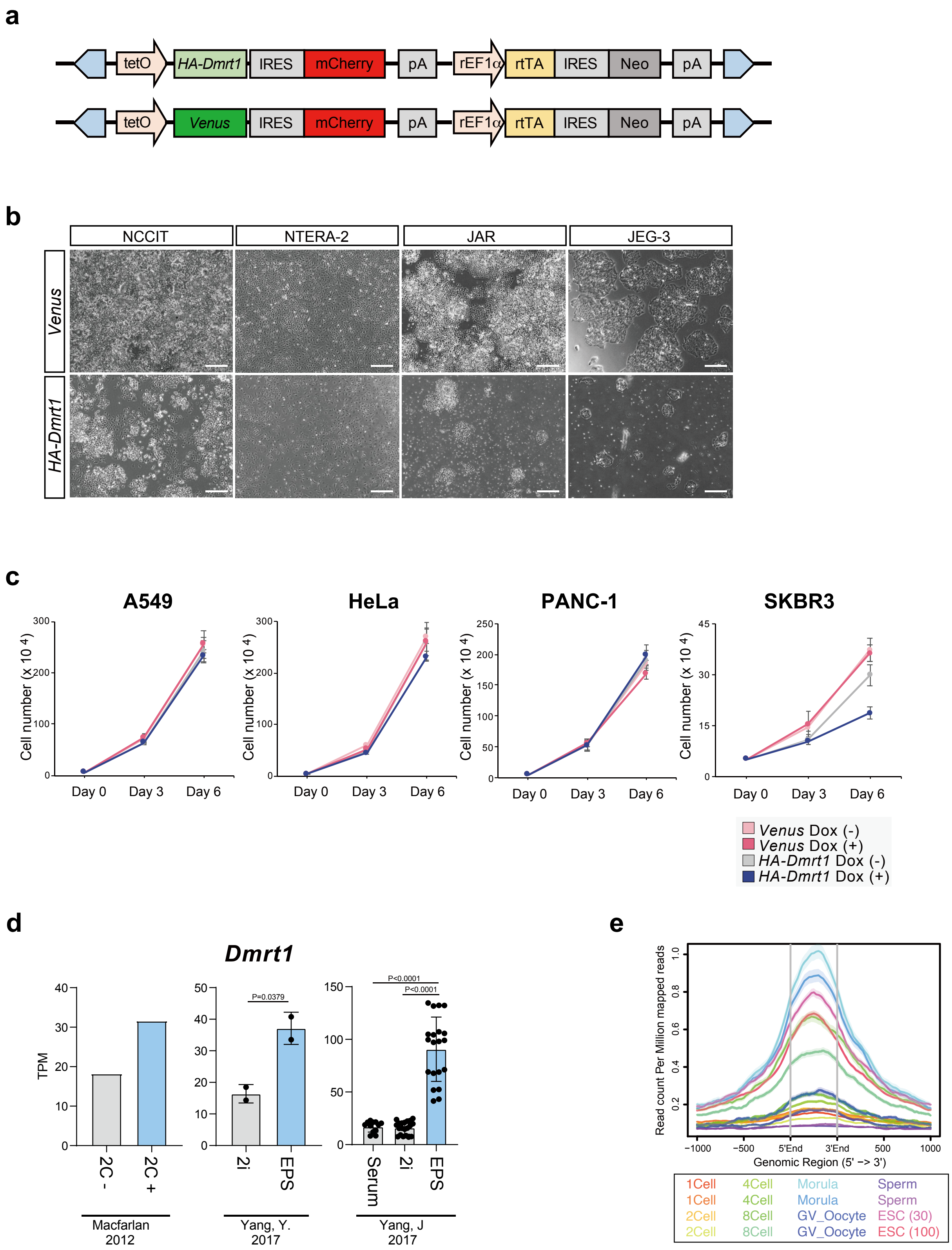

Supplementary Fig. 10: DMRT1-mediated reprogramming provides a unique therapeutic target for human germ cell tumors.

a, Schematic illustration of the *piggyBac* vector construct for Dox-inducible *Dmrt1* or *Venus*.
b, Representative images of human GCT cell lines carrying Dox-inducible *Dmrt1* or *Venus* alleles after Dox treatment (NCCIT, Day 5; NTERA-2, Day 6; JAR, Day 6; JEG-3, Day 6). Scale bars: 500 μm.
c, Cell proliferation assay in *Dmrt1*-expressing human cancer cell lines (A549: lung adenocarcinoma; HeLa: cervix epithelioid carcinoma; PANC-1: pancreas ductal adenocarcinoma; SKBR3: breast adenocarcinoma). Data are presented as means ± SD of three independent experiments.
d, Expression levels of *Dmrt1* in previously reported mouse PSCs with expanded differentiation potential. RNA-seq data of PSCs were obtained from GSE33923 (2C::tomato-, n=1; 2C::tomato+, n=1), GSE89303 (2i, n=2, EPS, n=2) and ERP005641 (Serum, n=12; 2i, n=20; EPS, n=20). Data are presented as means ± SD (Yang, Y., 2017 [6]: t-test, two-sided; Yang, J., 2017 [5]: Kruskal–Wallis test and Dunn's multiple comparisons test, two-sided.).
e, DNase-seq analysis for DMRT1 binding sites in E13.5 gonadal cells in preimplantation embryos at various developmental stages. The DMRT1 binding sites were the most accessible at the morula stage during preimplantation embryogenesis. Shading represents the standard error of the mean (SEM).

Reference

1. Wu, J.Y. *et al.* The landscape of accessible chromatin in mammalian preimplantation embryos. *Nature* **534**, 652-+ (2016).
2. Nakamura, T. *et al.* A developmental coordinate of pluripotency among mice, monkeys and humans. *Nature* **537**, 57-62 (2016).
3. Abad, M. *et al.* Reprogramming in vivo produces teratomas and iPS cells with totipotency features. *Nature* **502**, 340-5 (2013).
4. Macfarlan, T.S. *et al.* Embryonic stem cell potency fluctuates with endogenous retrovirus activity. *Nature* **487**, 57-+ (2012).
5. Yang, J. *et al.* Establishment of mouse expanded potential stem cells. *Nature* **550**, 393-397 (2017).
6. Yang, Y. *et al.* Derivation of Pluripotent Stem Cells with In Vivo Embryonic and Extraembryonic Potency. *Cell* **169**, 243-257 (2017).
7. Korkola, J.E. *et al.* Down-regulation of stem cell genes, including those in a 200-kb gene cluster at 12p13.31, is associated with in vivo differentiation of human male germ cell tumors. *Cancer Research* **66**, 820-827 (2006).

Table S1. Primer used in this study.

qPCR	Sequences (5'→3')	
	Forward	Reverse
Mouse: Actb	GCCAACCGTGAAAAGATGAC	TCCGGAGTCCATCACAATG
Mouse: Pou5f1	TAGGTGAGCCGTCTTTCCAC	GCTTAGCCAGGTTCGAGGAT
Mouse: Sox2	CGTAAGATGGCCCAGGAGAA	GCTTCTCGGTCTCGGACAAA
Mouse: Klf4	GACTAACCGTTGGCGTGAGGA	CTGCAAGCTGCACCAGCTC
Mouse: Myc	CACCAGCAGCGACTCTGA	GGGGTTTGCCTCTTCTCC
Mouse: Nanos3	CACTACGGCCTAGGAGCTTGG	TGATCGCTGACAAGACTGTGG
Mouse: Dazl	TCCTTGACTTGTGGTTGCTG	CCACCTTCGAGGTTTTACCA
Mouse: Dppa3	AGGCTCGAAGGAAATGAGTTTG	TCCTAATTCTTCCCGATTTTCG
Mouse: Dmrt1	GAAGACCTCAGAGAGCCGCC	CCTTCCTCGAAGGTAAAGATCTGGG
Mouse: mCherry	CCCGCCGACATCCCCGACTA	GGGTCACGGTCACCACGCC
Mouse: Ddx4	TATGTGCCTCCCAGCTTCAGTA	CTGGATTGGGAGCTTGTGAAGA
Mouse: Nanog	TGCTTACAAGGGTCTGCTACTG	TAGAAGAATCAGGGCTGCCTTG
Mouse: Prdm14	ACAGCCAAGCAATTTGCACTAC	TTACCTGGCATTTTCATTGCTC
Mouse: Dnd1	CCCTAAATGGGTTAAGCAGAGC	GGCAAGGTTCCTCACAACTAAAG
Mouse: Cdx2	CAAGGACGTGAGCATGTATCC	GTAACCACCGTAGTCCGGGTA
Mouse: Prdm1	GCCACAAGAGATTTAGCAGCACAA	CAGTCGCTTGTGCAGCTTCAG
Mouse: Hand1	GCTCCACGAACCCTTCCTGT	CAGCAGCCAGCTCTGGAAGTA
Mouse: Ascl2	GAGCAGGAGCTGCTTGACTT	CAGTCAGCACTTGGCATTTG
Mouse: Elf5	GACCAAGCGCCATGTCTGG	CAGGCCGCTGATGTTGAAGTG
Mouse: Eomes	GATGTACGTTCACCCAGAATCTCCT	CATCTGTGTGTTGTTGTTGTTTGCAC
Mouse: Tfap2c	GGACAACATCTCTACAGTCCCGCGC	CGAGTATGCCAGCTGCTGGTA
Human: GAPDH	ATGGGGAAGGTGAAGGTCG	GGGGTCATTGATGGCAACAATA
Human: GATA3	TGCAGGAGCAGTATCATGAAGCCT	GCATCAAACAACTGTGGCCAGTGA
Human: GATA2	GCACCTGTTGTGCAAATTGTCAGA	CTGGATCCCTTCCTTCTTCATGGT
Human: KRT7	GCGTGAGTACCAGGAACTCATG	GCTTGCGGTAGGTGGCG
Human: HLA-G	GCTGTGATCACTGGAGCTGT	ACTCTTGCCTCTCAGTCCCA
Human: CGB	CAACACCACCATCTGTGC	CTTTATTGTGGGAGGATC
Human: p16 (INK4A)	GTGCCACATTCGCTAAGTGCTC	CGACCCTGTCCCTCAAATCCTC
Human: p15 (INK4B)	GTCATGATGATGGGCAGCGC	CAGCATCATGCACCGGTCG
Human: p18 (INK4C)	GATTTGGAAGGACTGCGCTGC	CATGAATGACAGCGAAACCAGTTCG
Human: p19 (INK4D)	GCTTTCTGGCAGCTGAATCTGATC	CTCTTGCTGGAGAGGGTGACC
Human: p21 (CIP1)	GACCATGTGGACCTGTCACTGT	CCGTTTTCGACCCTGAGAGTCT
Human: p27 (KIP1)	GAGCAATGCGCAGGAATAAGGAA	CTCCACAGAACCGGCATTTGG
Human: p57 (KIP2)	GCGGCGATCAAGAAGCTGTC	CTGCTCCACCGAGCCCA

Bisulfite sequencing	Sequences (5'→3')	
	Forward	Reverse
H19 DMR (Outside)	GAGTATTTAGGAGGTATAAGAATT	ATCAAAAACTAACATAAACCCCT
H19 DMR (Inside)	GTAAGGAGATTATGTTTATTTTTGG	CCTCATTAATCCCATAACTAT
Nespas-Gnasxl DMR	GGTTTTGGGTTTTAGTTTTTGATTATTTA	TCTTCTAAAAATCCACTAATCTCACTACC

Cloning	Sequences (5'→3')	
	Forward	Reverse
Cre-pA-PGK-Bsd-pA (Pax8)	GTAGGAAAGCTGCGAGTGTCCCTCAGTCTGTGAGCGACTCCCCGGCGATGCCCAAGAAGAAGAGGAAGGT	GGGGTCCTGGCCCCTCCGCGGTCCTTACCGGATCTGATCGAGTTGTGAGGCTAACTTTAAATAATGCCAATTATTTAAAGTTAT
Cre-pA-PGK-Bsd-pA (Pdx1)	GCTTGTAGCTCCGACCCGGGGCTGCTGGCCCCCAAGTGCCGGCTGCCACCATGCCCAAGAAGAAGAGGAAGGTGT	CACGGGTCCTTGTAGAGCTGTGTGGCCGCGTAGTACTGCTCCTCACTGTTCTAACTTTAAATAATGCCAATTATTTAAAGTTATGAATTCAGACATG
Oct4-P2A-Sox2-T2A-Klf4-E2A-c-Myc with Kozak sequence	GCCACCATGGCTGGACACCTGGCTTC	TTATGCACCAGAGTTTCGAAGCTG
Venus with Kozak sequence	GCCACCATGGTGAGCAAGGGCGAGG	TTACTTGTACAGCTCGTCCATGCC
P2A-CreERT2-pA-PGK-Bsd-pA (Nanog)	AAGCCTTGGAATTATTCCTGAACTACTCTGTGACTCCACCAGGTGAAATAGCcAGCGGCAGCGG	AGGAACCTGGCTTTGCCCTGACTTTAAGCCCAGATGTTGCGTAAGTCTCAAGACATGATAAGATACATTGATGAGTTTGGACA
HA-Dmrt1	ATGTACCCCTACGACGTGCCCGACTACGCCCCGAACGACGACACATTCG	TCACTCGTCCTCATCCTCTTCC
Dmrt1 with Kozak sequence	GCCACCATGCCGAACGACGACACATTC	TCACTCGTCCTCATCCTCTTCC
HA-Cdx2	ATGTACCCCTACGACGTGCCCGACTACGCCTACGTGAGCTACCTTCTGGACAAG	TCACTGGGTGACAGTGGAGTTT

Knockout experiments	Sequences (5'→3')	
	Forward	Reverse
Cdx2	GTTTGTCCCCAGACAGCATGG	CATCCACTCGCACAGGTTTCG
Tead4	GTTTATACCCCTGGGTTCCCACA	CCAGGTTGGCCTGGCAAAG
Dmrt1	GTACAACCCCCCACCCACT	CTGCACTTCTTGCACTGGCA

ATAC-qPCR	Sequences (5'→3')	
	Forward	Reverse
Rpl12	ACCTCTGGTGCAGCCCTAC	GAGCTTGGTCTGTGGGTGAG
Chr11	ACAGGAAAAACTCAAAAGTCCATCC	TCCTTTCCCAGCCTTGAGTT
Chr4	CATGCGGTAATGCTCAGCAC	AGGATGGTCTCTGCCTGCTA
Nanog	GGCAGCAAGGTCTGACTCTT	CTTTTAACCACGGCTGCACC
