# Supplementary material for: DMRT1-mediated reprogramming drives development of cancer resembling human germ cell tumors with features of totipotency
Source: Nat Commun. 2021 Aug 19;12:5041. doi: 10.1038/s41467-021-25249-4 (PMC8377058; doi:10.1038/s41467-021-25249-4)
Supplement: Supplementary file 3 — Reporting summary [file 41467_2021_25249_MOESM3_ESM.pdf]

nature research

Corresponding author(s): Yasuhiro Yamada
Last updated by author(s): Jul 20, 2021

Reporting Summary

Nature Research wishes to improve the reproducibility of the work that we publish. This form provides structure for consistency and transparency in reporting. For further information on Nature Research policies, see our Editorial Policies and the Editorial Policy Checklist.

Statistics

For all statistical analyses, confirm that the following items are present in the figure legend, table legend, main text, or Methods section.

n/a	Confirmed	
☐	☒	The exact sample size (*n*) for each experimental group/condition, given as a discrete number and unit of measurement
☐	☒	A statement on whether measurements were taken from distinct samples or whether the same sample was measured repeatedly
☐	☒	The statistical test(s) used AND whether they are one- or two-sided<br>*Only common tests should be described solely by name; describe more complex techniques in the Methods section.*
☐	☒	A description of all covariates tested
☐	☒	A description of any assumptions or corrections, such as tests of normality and adjustment for multiple comparisons
☐	☒	A full description of the statistical parameters including central tendency (e.g. means) or other basic estimates (e.g. regression coefficient) AND variation (e.g. standard deviation) or associated estimates of uncertainty (e.g. confidence intervals)
☐	☒	For null hypothesis testing, the test statistic (e.g. *F*, *t*, *r*) with confidence intervals, effect sizes, degrees of freedom and *P* value noted<br>*Give P values as exact values whenever suitable.*
☒	☐	For Bayesian analysis, information on the choice of priors and Markov chain Monte Carlo settings
☒	☐	For hierarchical and complex designs, identification of the appropriate level for tests and full reporting of outcomes
☒	☐	Estimates of effect sizes (e.g. Cohen's *d*, Pearson's *r*), indicating how they were calculated

Our web collection on statistics for biologists contains articles on many of the points above.

Software and code

Policy information about availability of computer code

Data collection

RNA sequencing
RNA (in vitro and in vivo samples; 200 ng, FACS-sorted mCherry-positive tumor cells; 50 ng) was prepared for library construction. RNA-seq libraries were generated using the TruSeq Stranded mRNA LT sample prep kit (Illumina) or NEBNext Ultra II Directional RNA Library Prep kit for Illumina (NEB). The libraries were sequenced on a NextSeq500 (Illumina) as 86 bp single reads.

Whole-genome bisulfite sequencing (WGBS) and Methyl-sequencing
500ng of DNA was fragmented by sonication (Covaris) and ligated with methylated adapters using TruSeq™ Nano DNA Library Prep Kit and TruSeq™ ChIP Libary Preparation Kit (Illumina). Subsequently, DNA was bisulfite-treated using EZ DNA Methylation-Gold Kit™ (ZYMO RESEARCH). Final library amplification was performed using Pfu Turbo Cx (Agilent Technologies). For methyl-seq library, 1μg of DNA was fragmented by sonication. Subsequently, library preparation was performed using the SureSelectXT Mouse Methyl-Seq Reagent Kit (Agilent Technologies). DNA was bisulfite-treated using the EZ DNA Methylation-Gold Kit™. The libraries were then sequenced on a Hiseq 2500 (Illumina) in a rapid mode as paired-end reads (Read1:109 bp, Read2:108bp).

Assay for transposase-accesible chromatin sequencing (ATAC-seq)
Cells were lysed with a lysis buffer (10mM Tris-HCl pH7.4, 3mM MgCl2, 10mM NaCl and 0.1% IGEPAL CA-630). Yielded nuclei were suspended in the transposase reaction mix (25 μl of 2X TD Buffer (Illumina), 2.5 μl of TD Enzyme 1 (Illumina), and 22.5 μl of nuclease-free water), and incubated for 30 minutes at 37 ℃. Fragmented DNA was then purified using MinElute PCR purification kit (QIAGEN). After that, fragmented DNA was amplified by PCR to obtain ATAC-seq libraries. The libraries were paired-end sequenced with a length of 50 bp on Hiseq 1500 (Illumina).

Data analysis

For RNA-seq analysis, after trimming of adaptor sequences and low-quality bases with cutadapt-1.18, sequenced reads were mapped to the mouse reference genome (mm10) and expression levels were calculated as TPM using RSEM 1.3.1 with STAR (v. 2.6.0c) aligner and the

GENCODE version M23 annotation gtf file.

For WGBS and MethylC-seq analyses, bases with low-quality scores and the adapters in all sequenced reads were trimmed with cutadapt-1.18. Trimmed reads were mapped to mouse reference genomes (mm10) using the Bismark software-v0.20.1 with bowtie2 (version 2.3.5). Methylated cytosines were extracted from reads using the Bismark methylation extractor with the following options: --ignore 10 --ignore_r2 10 --ignore_3prime 5 --ignore_3prime_r2 5.

For ATAC-seq analysis, adaptor sequences in reads were trimmed using Trim Galore (version 0.3.7.) Then trimmed reads were aligned to the mouse reference genome (mm10) build using bowtie (version 0.12.7) with default parameters. Duplicated reads were removed using samtools (version 0.1.18).

For manuscripts utilizing custom algorithms or software that are central to the research but not yet described in published literature, software must be made available to editors and reviewers. We strongly encourage code deposition in a community repository (e.g. GitHub). See the Nature Research guidelines for submitting code & software for further information.

Data

Policy information about availability of data

All manuscripts must include a data availability statement. This statement should provide the following information, where applicable:

- Accession codes, unique identifiers, or web links for publicly available datasets
- A list of figures that have associated raw data
- A description of any restrictions on data availability

All data of WGBS, Methyl-seq, RNA-seq and ATAC-seq used in this study were deposited in the Gene Expression Omnibus (GEO) under accession number GSE149499.
The publicly available datasets used in this study are: GSE63392 (Gkountela et al., 2015), GSE92280 (Guo et al., 2017), GSE3218 (Korkola et al., 2006), GSE66390 (Wu et al., 2016), GSE33923 (Macfarlan et al., 2012), GSE48364 (Abad et al., 2013), GSE89303 (Yang et al., 2017b), ERP005641 (Yang et al., 2017a)

Field-specific reporting

Please select the one below that is the best fit for your research. If you are not sure, read the appropriate sections before making your selection.

☒ Life sciences ☐ Behavioural & social sciences ☐ Ecological, evolutionary & environmental sciences

For a reference copy of the document with all sections, see nature.com/documents/nr-reporting-summary-flat.pdf

Life sciences study design

All studies must disclose on these points even when the disclosure is negative.

Sample size	No statistical methods were used to predetermine sample size. Sample size was chosen based on standards in the field. Sample size is described in methods, figures or figure legends. qRT-PCR experiments were performed in biological triplicate. ATAC-qPCR experiments were performed in biological duplicate. We have had enough number of sequencing reads for WGBS, Methyl-seq, RNA-seq and ATAC-seq.
Data exclusions	Since we used chimeric mice to evaluate pathological data, we excluded mice with no chimeric contribution. We also excluded a few mice which unexpectedly died during Dox treatment. We didn't excluded any other data from the analysis to avoid the arbitrary selection.
Replication	We were able to confirm the reproducibility of our results. See experimental materials and methods section.
Randomization	In animal experiments, chimeric mice used were allocated to arrange coat color chimerism to avoid the effect of chimerism. For quantification of histological features, HE-stained sections and immunostained sections were randomly photographed at 100× magnification. Four or five images from each sample were processed with ImageJ software (NIH) to evaluate the region of interest.
Blinding	We were not blinded during data collection and analysis. We did not considered blinding required in these type of experiments.

Reporting for specific materials, systems and methods

We require information from authors about some types of materials, experimental systems and methods used in many studies. Here, indicate whether each material, system or method listed is relevant to your study. If you are not sure if a list item applies to your research, read the appropriate section before selecting a response.

Materials & experimental systems

n/a	Involved in the study
☐	☒ Antibodies
☐	☒ Eukaryotic cell lines
☒	☐ Palaeontology and archaeology
☐	☒ Animals and other organisms
☒	☐ Human research participants
☒	☐ Clinical data
☒	☐ Dual use research of concern

Methods

n/a	Involved in the study
☒	☐ ChIP-seq
☐	☒ Flow cytometry
☒	☐ MRI-based neuroimaging

Antibodies

Antibodies used

For immunostaining and immunocytochemistry:
mouse monoclonal anti-OCT4 (BD, #611203, dilution 1/200), rabbit monoclonal anti-NANOG (Cell Signaling Technology, Cat#8822, dilution 1/1000), rabbit monoclonal anti-Ki67 (Abcam, #ab16667, dilution 1/200), rabbit polyclonal anti-2A peptide (Merck Millipore, #ABS31, dilution 1/250), rabbit monoclonal anti-DAZL (Abcam, #ab215718, dilution 1/200), rabbit monoclonal anti-CDX2 (Thermo Fisher Scientific, #MA5-14494, dilution 1/200), mouse monoclonal anti-AP-2γ (Tfap2c; Santa Cruz Biotechnology, #sc-12762, dilution 1/200), goat polyclonal anti-Placental lactogen Ⅰ (PL-1; Santa Cruz Biotechnology , #sc-34713, dilution 1/100), rabbit monoclonal anti-GFP (Abcam, #ab183734, dilution 1/200), CF488A anti-mouse IgG (Biotium, #20014, dilution 1/500), CF555 anti-rabbit IgG (Biotium, #20038, dilution 1/500), . Alexa Fluor 647 anti-rabbit IgG (Invitrogen, #D21490, dilution 1/750)

For Wetsern blot:
mouse monoclonal anti-OCT4 (BD, #611203, dilution 1/500), mouse monoclonal anti-SOX2 (Merck Millipore, #MAB4343, dilution 1/100), rabbit polyclonal anti-KLF4 (Thermo Scientific, #PA5-27440, dilution 1/300), rabbit monoclonal anti-c-MYC (Abcam, #ab32072, dilution 1/100), mouse monoclonal anti-TEAD4 (Abcam, #ab58310, dilution 1/100), mouse monoclonal anti-DMRT1 (Santa Cruz Biotechnology, #sc-377167, dilution 1/100), mouse monoclonal anti-GAPDH (Invitrogen, #AM4300, dilution 1/1000), sheep anti-mouse IgG (GE HealthCare, #NA931, dilution 1/1000), donkey anti-rabbit IgG (GE HealthCare, #NA934, dilution 1/1000).

Validation

Validation of dilution was determined in accordance with manufacturer's directions. In some primary antibodies, we optimized the dilution to fit our staining protocols.

Eukaryotic cell lines

Policy information about cell lines

Cell line source(s)

The KH2 OSKM and LacZ ESC line was described previously (Ohnishi et al., 2014). Other mouse ES cell lines and MEFs were established in our laboratory. Human cancer cell lines were NCCIT, NTERA-2 a1.D1, JAR, JEG-3, SKBR3 were obtained from ATCC (CRL-2073, CRL-1973, HTB-144, HTB-36, HTB-20). A549 and HeLa were obtained from RIKEN (RBRC-RCB0098, RBRC-RCB0007). PANC-1 was obtained from DS pharma biomedical (EC87092802-F0).

Authentication

We authenticated that each cell lines have correct genotypes using PCR, immunostaining and western blot.

Mycoplasma contamination

All samples used were tested for mycoplasma contamination. We confirmed that our cell lines are negative to mycoplasma contamination.

Commonly misidentified lines
(See ICLAC register)

No commonly misidentified lines were used in this study.

Animals and other organisms

Policy information about studies involving animals; ARRIVE guidelines recommended for reporting animal research

Laboratory animals

We performed in vivo reprogramming experiments using 4-8 week old chimeric mice. In pathological investigations, we used both male and female mice. Pseudopregnant ICR and male/female ICR (8-10 week old) mice were obtained from Japan SLC to make chimeric mice. C57BL/6 mice were also obtained from Japan SLC. BALB/c-nu/nu mice were obtained from Japan CLEA.

Wild animals

No wild animals were used in this study.

Field-collected samples

No field-collected samples were used in this study.

Ethics oversight

All experiments using animals were performed under the ethical guidelines of Kyoto University and The University of Tokyo.

Note that full information on the approval of the study protocol must also be provided in the manuscript.

Flow Cytometry

Plots

Confirm that:

- [x] The axis labels state the marker and fluorochrome used (e.g. CD4-FITC).
- [x] The axis scales are clearly visible. Include numbers along axes only for bottom left plot of group (a 'group' is an analysis of identical markers).
- [x] All plots are contour plots with outliers or pseudocolor plots.
- [x] A numerical value for number of cells or percentage (with statistics) is provided.

Methodology

Sample preparation	For preparation of in vitro samples, cells were washed with PBS and incubated in 0.25% trypsin-EDTA (Nacalai Tesque) for 5 min at 37°C. For in vivo tumor samples, dissociation of tumor tissues was performed as described above. Cell pellets were resuspended in FACS buffer (PBS containing 4% BSA) and passed through a cell strainer. GFP- or mCherry-positive cells were sorted by FACS Aria-II or Aria-III (BD).
Instrument	FACS AriaII and Aria-III (BD)
Software	FlowJo software (FlowJo, LLC; https://www.flowjo.com)
Cell population abundance	Abundance of distinct cell populations of interest was determined using appropriate negative controls and puritiy of sorted populations as determined by post sort reanalysis.
Gating strategy	Standard gating setting commonly utilized at flowcore facility of The University of Tokyo were used. Cell debris was excluded using a FSC and SSC gate. Dox-untreated samples (negative controls) were used to set appropriate gates.

- [x] Tick this box to confirm that a figure exemplifying the gating strategy is provided in the Supplementary Information.
